# Comparison of Bead-Based Fluorescence Versus Planar Electrochemiluminescence Multiplex Immunoassays for Measuring Cytokines in Human Plasma

**DOI:** 10.3389/fimmu.2020.572634

**Published:** 2020-09-24

**Authors:** Anna Günther, Matthias Becker, Jens Göpfert, Thomas Joos, Nicole Schneiderhan-Marra

**Affiliations:** NMI Natural and Medical Sciences Institute at the University of Tübingen, Tübingen, Germany

**Keywords:** cytokine, multiplex immunoassay, chemokine, dynamic range, lower limit of quantification, Meso Scale Discovery, Luminex, Millipore

## Abstract

This study compared two 96-well multiplex immunoassay platforms for analytical performance in assessing cytokine concentrations in standards, quality controls and human plasma samples (*n* = 62), and evaluated assay time requirements. Assays included a bead-based fluorescence MILLIPLEX^®^ assay/Luminex fluorescence platform (LMX) and three kits from Meso Scale Discovery (MSD) in planar electrochemiluminescence format. The LMX kit evaluated 21 cytokines and the MSD kits evaluated 10 cytokines each, with 16 overlapping cytokines between platforms. Both assays provided good reproducibility in standard curves for all analytes. Interassay CVs of shared analytes showed average kit quality control CVs ranging 1.9–18.2% for LMX and 2.4–13.9% for MSD. The MSD platform had lower LLoQs than LMX for 14/16 shared cytokines. For IL-17, the LLoQ was lower with LMX than MSD, and the LLoQs for IL-6 were similar. Although MSD calibration curves indicated lower LLoQs for most of those analytes, many more cytokines in human plasma samples were not detected by MSD than by LMX. The ULoQs were higher in LMX versus MSD assays for 13/16 shared analytes, lower than MSD for IL-17, and equivalent between assays for IL-6 and MIP-1α. Bland-Altman plots indicated that MSD classified 13/16 shared analytes as concentrations lower than by LMX. Time and motion analysis indicated that total mean assay times were 20 h 28 m and 21 h 33 m for LMX and MSD, respectively, including an overnight (17 h) incubation. The MSD assays employed a manufacturer-approved overnight incubation instead of the standard 2-h incubation, which kit instructions suggest might increase detection sensitivity. Hands-on labor time averaged 1 h 37 m for LMX and 2 h 33 m for MSD. In summary, assay selection factors should include selection of specific markers of interest, time and cost considerations, and anticipated cytokine concentrations in prospective samples.

## Introduction

Cytokines and chemokines (chemotactic cytokines) constitute a growing group of diverse small (<40 kDa) secreted bioactive protein molecules that are essential in cross-communication between cells and tissues (1, 2). The simultaneous but balanced release of multiple cytokines is important for maintaining normal homeostasis, and is also an essential component of a well-regulated immune response following pathogen exposure, trauma, and in disease states. Multiplex immunological assays employ a variety of technologies, including planar chemiluminescence and bead-based immunocapture platforms, to simultaneously evaluate levels of multiple circulating proteins, including cytokines and other biomarkers (3). These multiplex immunoassays are useful for understanding the complex underlying biochemical mechanisms and interactions that occur during diverse disease states, have great potential for advancing epidemiological research, and are emerging as valuable clinical diagnostic and prognostic health assessment tools. This is highlighted by the steadily increasing number of multiplex proteomic assays that receive FDA approval for clinical application (4). Identifying disease-specific biomarker signatures in human plasma may be useful in diagnosing diverse health disorders including neoplastic, cardiovascular, pulmonary, metabolic, autoimmune, neurodegenerative, and infection/sepsis pathologies, among many others (5–12). This approach may be valuable in deciding best treatment options, tracking disease progression and response to therapy, and formulating prognoses (13).

Multiplex assays confer several advantages over singleplex immunoassays for obtaining the same cumulative information, including enhanced efficiency and reduced cost when concurrently measuring multiple analytes from a single sample, higher throughput, and provision of a more comprehensive biochemical profile that may facilitate the development of individualized medical treatments (3, 4). These comparative benefits of multiplex protein analysis are most apparent when assessing complex expression profiles in limited-availability or valuable small-volume samples, and when streamlining cost, labor, and time expenditures are essential considerations.

Different multiplex proteomic platforms have unique strengths, limitations, instrument requirements, and performance characteristics. The current study directly compared the analytical performance of two different multiplex immunoassay platforms in assessing cytokine concentrations in human plasma samples, and evaluated the associated hands-on time required for sample analysis using each method. These multiplex assays were evaluated because they are both routinely used in our core laboratory facility, and are commonly used by other researchers (3). Both assays employed a 96-well microtiter plate format. The first platform was a bead-based fluorescence assay, the MILLIPLEX^®^ MAP Human High Sensitivity T Cell Magnetic Bead Panel (Merck EMD Millipore, Billerica, MA, United States), run on the Luminex^®^ FLEXMAP 3D^®^ instrument (14). This detection instrument was introduced in 2009 and has improved sensitivity, broader dynamic range (>4.5 logs), and higher throughput from the predecessor Luminex 100/200^TM^ technology used in earlier studies (3). The LMX instrument requires 20 min to read a 96-well plate. The comparator platform was the electrochemiluminescence-based Meso Scale Discovery solid-matrix assay (MSD V-Plex^®^ kits; Meso Scale Diagnostics LLC, Rockville, MD, United States), using the MSD SECTOR Imager 6000 detection instrument for read-out (15). The Sector 6000 used by us was marketed until at least 2014, and was gradually replaced in the company product line with the MS S600, introduced in 2013; both instruments have identical dynamic ranges (6 logs) and fast 96-well plate reading times (70 s), according to manufacturer-provided specification sheets and user manuals. The Luminex/MILLIPLEX and Meso Scale Discovery systems and associated results are hereafter abbreviated as “LMX” and “MSD,” respectively.

The goal of this study was to comparatively evaluate the analytical performance characteristics of the two representative electrochemiluminescence and bead-based fluorescence multiplex platforms in determining concentration profiles of 16 overlapping cytokines in human plasma samples. Therefore, we have selected samples with different expected cytokine levels: healthy – low, diabetic – increased (9), high procalcitonin (PCT) – high (12). We also performed a workflow time-and-motion study of the two systems to compare time and labor input needed to assess the 16 cytokines that are evaluated by both assays. These findings may assist investigators in selecting the most appropriate multi-analyte detection system for their specific research needs, and aid in optimizing associated fiscal and labor expenditures.

## Materials and Methods

### Multiplex Assay Kits

This study compared two multi-analyte detection systems. The MILLIPLEX^®^ MAP Human High Sensitivity T Cell Magnetic Bead Panel (Product #HSTCMAG28SMPX21; Merck EMD Millipore), is a bead-based fluorescence immunoassay that simultaneously evaluates 21 analytes (fractalkine, GM-CSF, IFNγ, IL-1β, IL-2, IL-4, IL-5, IL-6, IL-7, IL-8, IL-10, IL-12 (p70), IL-13, IL-17A, IL-21, IL-23, ITAC, MIP-1α, MIP-1β, MIP-3α, and TNFα) in suspension (14). Because the MSD 96-well electrochemiluminescence immunoassay kits that we used were limited to assessing 10 cytokines per assay, we evaluated identical samples using three different kits to achieve reasonable overlap with the 21-plex LMX assay. The Pro-inflammatory Panel 1 (Product #K15049G) measures IFN-γ, IL-1β, IL-2, IL-4, IL-6, IL-8, IL-10, IL-12 (p70), IL-13, and TNF-α); the Cytokine Panel 1 (Product #K15050G) measures GM-CSF, IL-1α, IL-5, IL-7, IL-12/IL-23 (p40), IL-15, IL-16, IL-17A, TNF-β, and VEGF-A; and the Chemokine Panel 1 (Product # K15047G) measures eotaxin, eotaxin-3, IL-8, IL-8 HA, IP-10, MCP-1, MCP-4, MDC, MIP-1α, MIP-1β, and TARC (15). Taken together, the LMX and MSD assays measured 16 common cytokine analytes: GM-CSF, IFNγ, IL-1β, IL-2, IL-4, IL-5, IL-6, IL-7, IL-8, IL-10, IL-12 (p70), IL-13, IL-17, MIP-1α, MIP-1β, and TNFα. All kits were used before their stated expiration dates, and all assay steps were performed in accordance with manufacturer instructions. To allow maximum binding time of the analytes for both assays, a 17 h incubation time primary antibody incubation step was performed according to the kit manuals (14, 15). This reflects our use of the MSD-approved overnight incubation instead of the standard 2-h incubation, which kit instructions suggest might increase detection sensitivity.

### Quantitative Detection Systems

Analyte levels in the LMX microtiter plates were assessed using a FLEXMAP^®^ 3D instrument operated with xPONENT Software V4.2 (both from Luminex), and MSD plates were read on a SECTOR^®^ Imager 6000 operated with MSD Discovery workbench software v.3.0/4.0 (both from Meso Scale Diagnostics).

### Samples

Sixty-two EDTA-anticoagulated human plasma samples were evaluated by both assays. Samples comprised 20 from healthy donors, 21 from subjects who had previously been diagnosed with diabetes mellitus, and 21 from individuals with elevated PCT levels (>3 ng/mL), an indicator of sepsis (12, 16, 17). All samples were purchased from Central BioHub GmbH (Hennigsdorf, Germany), and were stored at −80°C until used. Analysis of these commercially available anonymous human plasma samples was exempt from IRB oversight requirements. Study performance complied with the tenets of the Declaration of Helsinki. Demographic information on sample donors is provided in Table 1.

**TABLE 1:** Demographics of plasma donors.

**Parameter**	**Healthy**	**Diabetic**	**High PCT**
*n*	20	21	21
Gender, male/female, *n*	5/15	11/10	12/9
Age, years, mean ± SD	43.1 ± 9.8	47.0 ± 15.7	66.2 ± 17.6
Age, years, range	26–60	20–79	23–87

### Experimental Overview

All assays using both platforms were performed by a single technologist with more than a decade of experience in performing multiplex immunoassays, for uniformity. Prior to analysis, all samples were thawed and filtered of potential particulate matter through 0.45-μm pore-size syringe filters, and were maintained at 4°C. In all instances, samples were run in duplicate and each assay plate contained recombinant standards for each analyte and quality control (QC) samples provided with the kits (2 for LMX and 3 for MSD). Calibration curves were established according to the user manuals using serial 4-fold dilutions of each kit’s stock analyte standards. Blank values were established using the sample diluent supplied with each immunoassay kit. All samples were diluted 1:4 using the provided sample diluent and processed following the recommendations of the kit providers.

### Assay Performance Characteristics

Multiplex immunoassays require the ability to generate meaningful calibration curves for a panel of heterogeneous markers across physiological concentrations, under standardized assay conditions. The quantitative accuracy of a multiplex assay is dependent on the cumulative quality of individual analyte calibration curves, which is in turn determined by reagent quality, appropriate curve-fitting procedures, assay precision (coefficient of variation, CV), analyte % recovery, and assay limits-of-quantification (LoQs) (4, 18, 19). We assessed assay precision, the lower limits of detection (LoD), the lower and upper limits of quantification (LLoQ and ULoQ, respectively), and the resulting dynamic ranges for cytokine detection with both assays. The mean value of 28 blank values from LMX plates and of 22 blank signals from the MSD assay were used to calculate the LoD, which was defined as the mean blank signal + 3 × SD. The LLoQ was defined as either the analyte LoD or the lowest measurable standard value, whichever was higher. The ULoQ was defined as the highest standard curve point in each kit.

### Comparative Evaluation of Cytokine Levels in Human Plasma

We quantified cytokine levels in diverse human plasma samples using both platforms, and compared measured values and those outside each assay’s dynamic range, for the 16 cytokines that both assays have in common. Bland-Altman plots were generated to evaluate cytokine measurement agreement between LMX and MSD (20). Heat maps were generated to colorimetrically visualize the respective protein expression levels of shared analyte concentrations in plasma samples when measured by the LMX versus the MSD assays.

### Time and Motion Study

We evaluated and compared the precise time allocations required for labor (operator hands-on and/or mandatory observation) and total times including incubations to evaluate a similar number of analytes by both assay methods, in 96-well plate formats. Time studies were conducted over 4 days wherein at least two independent experiments were evaluated for each assay platform to comprehensively delineate methods and processes of testing. The same technologist performed all multiplex assays, and experimental steps and tasks were timed and those data collated by an independent, impartial observer from Nexus (Plano, TX, United States), a third-party healthcare consulting firm.

### Data Analysis

Luminex data (mean fluorescence intensity) were exported from the instrument as csv-files. Analyte concentrations (pg/mL) were determined by back calculations to the standard curves using a multiparametric fit using Bio-Plex Manager software v.6.2 (Bio-Rad, Hercules, CA, United States). MSD data (electrochemiluminescence values) were evaluated (fitting, back-calculation) with the with MSD Discovery workbench software v.3.0/4.0 and the results were then exported as Excel worksheets. Data were analyzed using R statistical software v.3.3.1^1^ and Excel v.16 (Microsoft Inc., Redmond, WA, United States).

## Results

### Standard Curve Reproducibility

Both assays provided good reproducibility in their standard curves, for all analytes, as anticipated from the performance data cited in each user manual. The interassay CV generally only increased to ≥15% at the very lowest standard concentrations in both assays. In the LMX assay, the CV of the lowest standard point (above zero) was >25% in 8/16 (50%) of common analytes; in the MSD assay, the CV of the lowest standard point was >25% in 4/16 (25%) of common analytes (not shown). During analyses of shared analyte measurements, the range of average QC sample CV was 2.4–13.9% in the MSD QC set (high, medium, and low-end concentrations). With the LMX assays (high and low-end concentrations only), the average QC sample CV range was 1.9–18.2%, with 3/16 (19%) analytes registering CV >15%, at the low-end concentration-only in 2 analytes (IL-7 and MIP-1α) and at both QC concentrations in 1 analyte (IL-10).

### Analytical Limits of Detection and Quantification, and Dynamic Range

**FIGURE 1:**
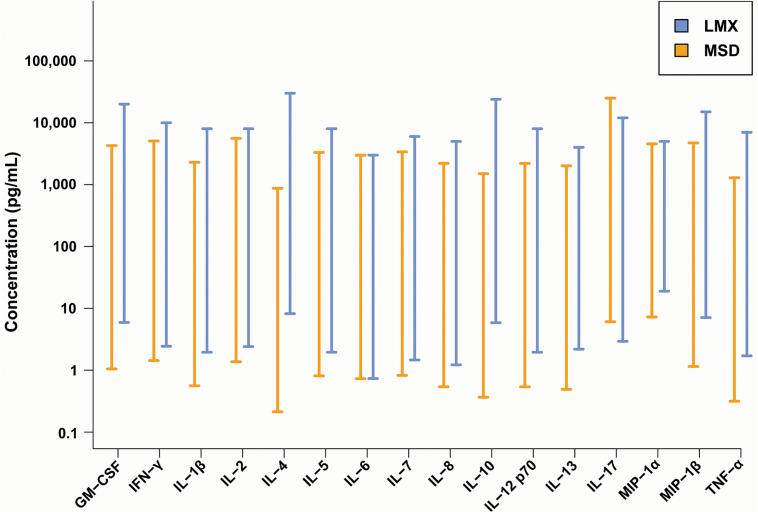
Dynamic ranges of the Luminex bead-based fluorescence (LMX) and Meso Scale Discovery electrochemiluminescence (MSD) multiplex cytokine immunoassay kits. With some exceptions (e.g., IL-6 and IL-17), the low-end of a shared analytes dynamic range was lower with the MSD compared to the LMX assay. Conversely, and with some exceptions (e.g., IL-6, IL-17, and MIP-1α), the high-end of the dynamic range was greater with the LMX assay versus the MSD assay. In two instances (IL-4 and IL-10), the differences in upper and lower quantification levels differed by at least an order of magnitude between the two assays.

**FIGURE 2:**
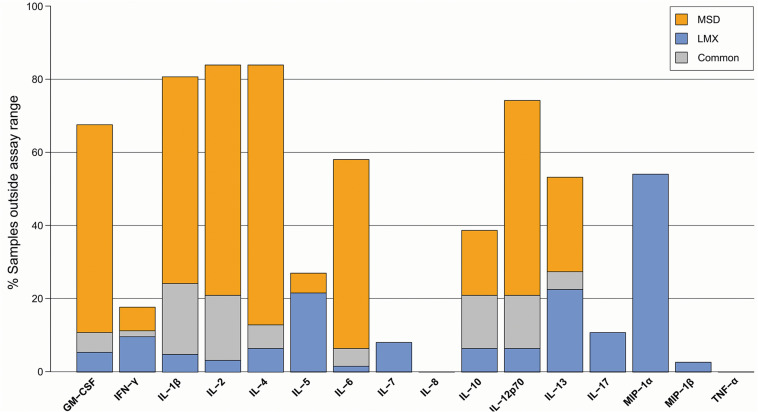
Proportion of donor plasma samples outside the assay quantification range for the Luminex (LMX) and Meso Scale Discovery (MSD) multiplex cytokine immunoassay kits. Although the MSD assay had a LLoQ below that of the LMX assay for 14/16 common analytes in earlier standard curve performance evaluations, this did not directly translate to evaluation of cytokines in experimental human plasma samples, whereby several analytes were missed at a greater frequency with the MSD platform (e.g., IL-10, IL-12, IL-1β, IL-2, IL-4, IL-6, and GM-CSF) and others were missed at a greater frequency with the LMX platform but detected with the MSD assay (e.g., IFN-γ, IL-5, IL-7, IL-17, MIP-1α, and MIP-1β).

The LoD and LLoQ of all analytes evaluated with each assay (detailed in Supplementary Table 1). The dynamic range was defined as the spread between the LLoQ and the highest standard curve point (ULoQ), and was determined for all 35 analytes coordinately and individually measured by the LMX and MSD systems (Supplementary Table 1 and Figure 1). The dynamic ranges of quantification for the 16 cytokine analytes that both assay platforms have in common are shown graphically in Table 2 and Figure 2. The LMX assay had higher ULoQs for 13 of 16 analytes, a slightly lower ULoQ for IL-17, and essentially identical ULoQs for IL-6 and MIP-1α, compared with the MSD assay. The MSD platform had lower analytical LLoQs than LMX for 14 of 16 shared cytokines. For IL-17, the LLoQ was lower in the LMX assay than with MSD, and the LLoQs for IL-6 were similar between platforms. In two instances (IL-4 and IL-10) the high and low quantification limits differed by more than an order of magnitude between assay systems. Relative size comparisons between the LMX and MSD assays’ dynamic ranges for each shared analyte are presented in Supplementary Table 2.

**TABLE 2:** Percentage of human plasma samples with target cytokines below the LLoD using the Luminex (LMX) and Meso Scale Discovery (MSD) multiplex assays.

**Shared analyte**	**LMX% Missed**	**MSD% Missed**	**LMX and MSD% Missed**
GM-CSF	5.4	**56.8**	5.4
IFN-γ	9.7	6.5	1.6
IL-1β	4.8	**56.5**	19.4
IL-2	3.2	**62.9**	17.7
IL-4	6.5	**71.0**	6.5
IL-5	21.6	5.4	0.0
IL-6	1.6	**51.6**	4.8
IL-7	8.1	0.0	0.0
IL-8	0.0	0.0	0.0
IL-10	6.5	17.7	14.5
IL-12 p70	6.5	**53.2**	14.4
IL-13	22.6	25.8	4.8
IL-17	10.8	0.0	0.0
MIP-1α	**54.1**	0.0	0.0
MIP-1β	2.7	0.0	0.0
TNF-α	0.0	0.0	0.0

*Bolded values signify that the target cytokine was undetected (below LLoD) in ≥50% of samples by the indicated platform.*

### Proportion of Samples Within Assay Quantification Range

A total of 62 plasma samples were evaluated using both platforms. Of the 16 common cytokine targets of both the LMX and MSD systems, some plasma samples had undetectable levels by either or both assays (Figure 2). Despite having lower LLoQs for a plurality of common analytes, in many instances the chemiluminescence assay was unable to detect cytokines in plasma samples that were quantifiable using the bead-based LMX platform (e.g., IL-10, IL-12, IL-1β, IL-2, IL-4, IL-6, and GM-CSF). Conversely, other cytokines were measurable in some plasma samples using MSD but were below the LLoQ of the LMX assay in a varying proportion of samples (e.g., IFN-γ, IL-5, IL-7, IL 17, MIP-1α, and MIP-1β).

**FIGURE 3:**
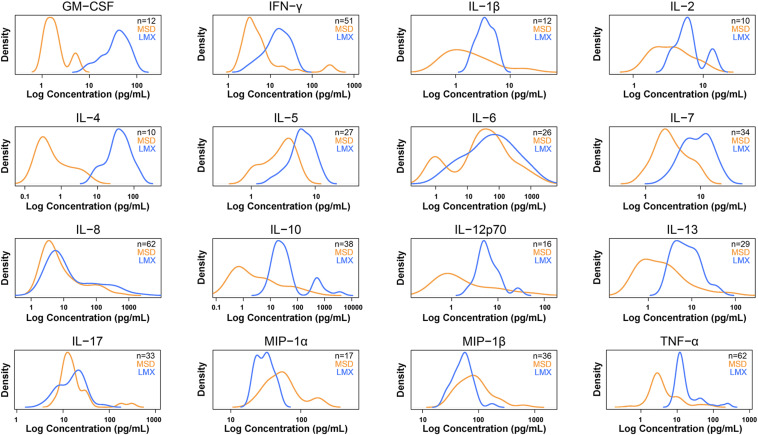
Density plots for 16 cytokines measured in human plasma samples by both methods that were within each assay’s dynamic range. Density plots show the distribution of the measured sample concentrations using a kernel density estimation. Luminex (LMX) is shown in blue and Meso Scale Discovery (MSD) in orange. Concentrations (*x*-axis) are on log scale. Density (*y*-axis) is scaled so that the total area under the two curves is the same. These plots give an overview of how sample measurement compares between the two methods.

Whereas six cytokines [IL-1β, IL-2, IL-4, IL-6, IL-12 (p70), and GM-CSF] were below quantifiable levels in ≥50% of samples using the MSD platform, only 1 analyte (MIP-1α) was not measurable in ≥50% of samples using LMX (Table 2). Additional information on the distribution of individual cytokine levels measured by the two assays is provided as signal density histograms in Figure 3.

### Agreement Analysis

**FIGURE 4:**
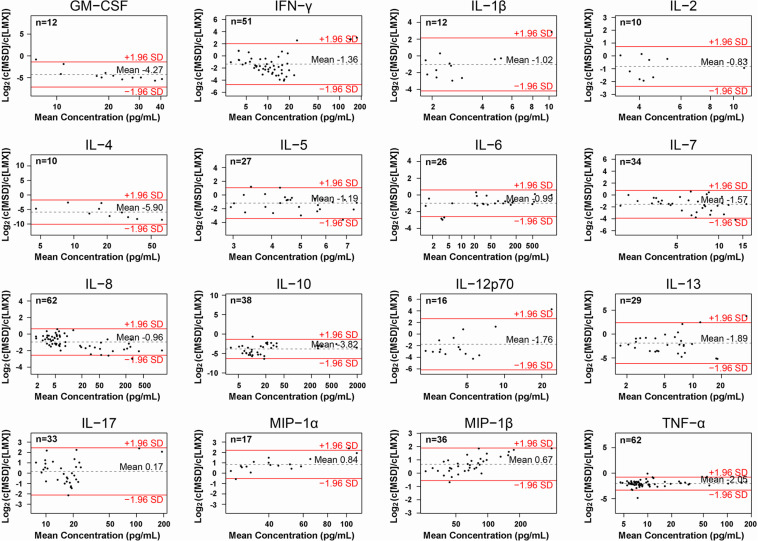
Agreement analysis of concentrations of the 16 cytokines measured in human plasma samples by the Luminex (LMX) and Meso Scale Discovery (MSD) multiplex assays, to assess agreement between the two platforms. Log_2_ values of the MSD/LMX concentration ratios are shown on the *y*-axis, and provide a visual assessment of estimates of systematic differences in measurement between methods when the mean value deviates from 0. In this presentation, a negative shift in the mean indicates that the MSD assay classifies the analyte as concentrations lower than LMX, and vice versa. Mean analyte concentrations (in pg/mL) are shown on the logarithmic *x*-axis, and precise values appear above the dotted horizontal “mean” lines in each graph. The ±1.96 SD lines represent the 95% limits of agreement, and when SDs are large or *n*-values are low, then interpretation can be ambiguous. In general, for most shared analytes, the mean is shifted to a negative value, indicating that MSD quantified an analyte at a lower concentration than LMX did using the same samples. Exceptions appear to be MIP-1α and MIP-1β, where LMX on average characterizes samples at lower concentrations. An example of where LMX and MSD are comparable is IL-17, where the mean ratio is close to 0. Proportional bias is suggested in the case of MIP-1β, wherein the mean concentration ratio approximates 0 at lower measured concentrations, but increases toward +2 at higher analyte concentrations.

Agreement between LMX and MSD multiplex assay measurement of the 16 common analytes evaluated in human plasma samples were visualized using Bland-Altman-based plots (Figure 4) (20). Log_2_ values of the mean MSD/LMX measured analyte concentration ratios shown on the *y*-axis provide estimates of systematic bias. Good agreement is presumed between assay methods if the mean value approximates 0 deviation. In this presentation, a negative shift in the mean indicates that the MSD assay classifies the analyte at concentrations lower than LMX, and vice versa. Mean analyte concentrations in each sample (pg/mL) are shown on the logarithmic *x*-axis. The ±1.96 SD lines represent the 95% limits of agreement. When SDs are large or *n*-values are low, graph interpretation can be ambiguous. For most shared analytes, the mean concentration ratio was shifted to a negative value, indicating MSD classification of target analyte in plasma samples at a lower concentration than LMX. Exceptions appear to be MIP-1α and MIP-1β, where LMX on average characterizes samples at higher concentrations. Agreement analysis also evaluates potential analyte-concentration-dependent effects (i.e., proportional bias), whereby agreement between the two assay methods changes as the analyte concentration in samples increases, as is suggested for MIP-1β. In this instance, the mean concentration ratio of the two assays is approximately 0 (no bias suggested) when samples with lower MIP-1β levels are assessed, but appears to increase toward +2 (bias toward LMX) at the highest measurable analyte concentrations.

### Heatmap Depiction of Relative Cytokine Concentrations Measured in Health and Disease

**FIGURE 5:**
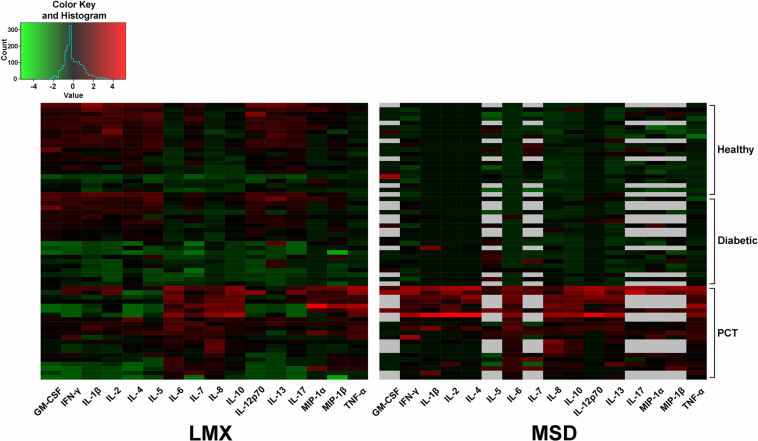
Heatmap depiction of relative analyte concentrations measured by the Luminex (LMX) and Meso Scale Discovery (MSD) multiplex assays. Cytokine measurements were performed in plasma samples from healthy individuals and from people with diabetes or elevated procalcitonin (PCT) levels. Data were centered and scaled per analyte per method. A histogram showing the color key used to designate sample concentrations is provided to the top left of the main image. Each cell represents a single analyte in a single plasma sample. Black cells indicate cytokine levels in that sample that were measured to be the approximate mean of all evaluated samples. Red cells indicate higher (above average) measured cytokine values for that sample/assay combination and green cells indicate lower (below average) analyte concentrations, with color intensity indicating magnitude of difference. Gray cells mean that sample data for that specific analyte were not available. Analyzed samples that provided signals below the lower limit of quantification (LLoQ) for any analyte had those values artificially entered as their specific LLoQ value before calculation and mapping.

A heatmap was generated to visually exemplify shared cytokine analyte measurements by LMX and MSD in diverse health states (Figure 5). Cytokine measurements were performed in plasma samples from healthy individuals and from people with diabetes or elevated PCT levels. A histogram showing the color key used to designate sample concentrations is provided to the top left of the main image. Each cell represents a single analyte in a single plasma sample. Black cells indicate cytokine levels in that sample that were measured to be the approximate mean of all evaluated samples. Red cells indicate higher (above average) measured cytokine values for that sample/assay combination and green cells indicate lower (below average) analyte concentrations, with color intensity indicating magnitude of difference. While sample analysis with either assay revealed a clear distinction in plasma cytokine levels between high-PCT subjects (indicative of sepsis) and both healthy and diabetic subjects, neither assay revealed clear expression pattern differences between samples from healthy versus diabetic subjects. Overall, there appeared to be poor agreement in cytokine expression profiles generated with the two assay systems; this effect may have been amplified by the fact that most cytokine levels measured in these samples were in the low end of both assays’ standard curves.

### Time and Motion Study

The total time required for performing the LMX 21-plex and three MSD 10-plex assays in 96-well format was recorded over several days (Supplementary Figure 1). The total time employed averaged 20 h 28 m and 21 h 33 m for the LMX and MSD assays, respectively, including an overnight (17 h) incubation for both assays. Of this time, mean hands-on labor time accounted for 1 h 37 m for LMX and 2 h 33 m for the MSD assays. Of notability is the required use of three MSD 96-well assays (maximum 10-plex) to overlap with most of the 21 cytokines evaluated on a single LMX 96-well plate. The MSD assays employed a manufacturer-approved overnight incubation instead of the standard 2-h incubation, which kit instructions suggest might increase detection sensitivity. Because comparison of MSD kit performance using the 2-h versus approved overnight sample incubations was beyond the scope of this study, we could not determine if the extended incubation indeed increased analyte detection sensitivity.

## Discussion

Serological quantification of individual circulating plasma protein levels using ELISA is an essential tool for both research and clinical investigations (21). However, cytokines rarely act alone but instead function in complex interactive networks (22, 23). Innovations in miniaturization, lithography, and fluidics have greatly expanded the development of multiplex immunoassay technologies, which have the advantage over singleplex ELISAs in that they can simultaneously quantify numerous bioactive molecules within a single small sample volume to give a more complete overview of circulating cytokines (4). This becomes particularly important with rare, expensive, or limited-volume samples.

Bead-based multiplex immunoassays incorporate collections of polymeric or magnetic microspheres (≈ 5–7 μm diameter) having different embedded fluorophores, coupled with unique cytokine-specific monoclonal antibodies that capture analytes in suspension. Binding of labeled detection antibodies allows bead isolation and quantification of targeted cytokines by flow cytometry (4, 14, 23). Planar multiplex electrochemiluminescence sandwich assays immobilize multiple analyte-specific capture antibodies to a solid matrix (slide or microtiter plate well). Bound target cytokines from samples are then recognized by detection antibodies coupled with electrochemiluminescence reporters that, when electrically stimulated, emit light whose wavelength and intensity correlates to the concentration of a specific analyte (6, 23).

This study compared the merits of two representative commercially available cytokine multiplex assay approaches, the MILLIPLEX magnetic bead-based fluorescence kit assayed on LMX instrumentation and the planar electrochemiluminescence MSD system, both in 96-well plate formats. Whereas multiplexing assays can in theory be designed to have large (e.g., ≥100) analyte-measuring abilities, this is generally not achievable due to antibody cross-reactivity reactions and other mitigating technical factors (4, 24). For example, the predisposition of liquid-phase multiplex assays to cross-reactivity increases in a quadratic relationship to the number of target analytes (25). The MILLIPLEX assay that we evaluated on the LMX platform simultaneously measures 21 cytokines. To arrive at acceptable overlap with the comparator MSD platform, we concurrently ran three different MDS 10-plex assay kits; 16 cytokines were coordinately assayed by both platforms.

Both intra- and inter-assay reproducibility, based on variance in respective kit QC sample and standard curve measurements, were similarly acceptable with both platforms. Excessive interassay CV variation (>15%) was only observed at the lowest standard curve concentration with both platforms for the 16 shared analytes. Quality control samples (2 in MILLIPLEX kits and 3 in MSD kits) generated appropriately reproducible signals with both platforms. Interassay CV for QC samples exceeded 15% for 4 shared analytes with the LMX platform, with a maximum observed value being 18.2%. The maximal average interassay CV for any shared analyte in any QC sample using the MSD platform was 13.9%. This is consistent with prior findings of high intra-assay reproducibility and comparable inter-assay precision between LMX and MSD technologies when measuring high analyte concentrations, but variable inter-assay variability at lower analyte concentrations that was analyte-dependent (26).

The dynamic range of fluorescence and electrochemiluminescence multiplex immunoassays is generally several orders of magnitude greater than that of single-plex colorimetric ELISAs (4, 27). In general, the LMX assay LLoQ was higher than with MSD for most shared analytes (14 of 16 cytokines), was approximately equal for one analyte (IL-6), and was lower than MSD for one analyte (IL-17). When considering the ULoQ, this pattern remained, with LMX showing a higher ULoQ than MSD for 13/16 cytokines, a lower ULoQ for IL-17, and approximately equivalent ULoQs for IL-6 and MIP-1α. Compared as ratios, both assays had similarly sized (within 20%) dynamic ranges for most (13/16) shared analytes, and MSD had a larger dynamic range than LMX for (approximately 2-fold) for 3 analytes: IL-13, MIP-1α, and MIP-1β. However, these internal performance characteristics did not directly translate over to measurements of cytokines in human plasma samples derived from a mixed group of healthy, diabetic, and high-PCT subjects. Despite the MSD assays having a lower LLoQ than LMX for most shared analytes on calibration curves, they were unable to detect a notably greater number of cytokines in actual plasma samples, and at a greater frequency, than the LMX platform. This was analyte-dependent, evidenced by the fact that in some instances the LMX assay missed detection more frequently than MDS. In one instance, IL-4, MSD displayed a LLoQ that was more than an order of magnitude lower than that of LMX in calibration curves. However, MSD failed to detect IL-4 in ≈70% of human plasma samples, whereas LMX, having a much less sensitive low-end detection capability for this molecule (based on calibration curves), was unable to detect IL-4 in only ≈10% of plasma samples.

Discrepancies in calibration curve and QC findings versus cytokine measurements in actual human samples could be due to several factors that can affect multiplex immunoassay sensitivity and specificity. Some of these parameters include variable cross-reactivity and interference across manufacturer-specific cytokine-detecting antibody pairs, differences in ligand binding to assay-specific antibodies, varying signal emission in different biological matrices, effects of kit-specific dilution buffers, and divergent antibody binding characteristics to kit-supplied recombinant protein standards versus native proteins (3, 4, 23, 24). In earlier versions of the LMX and MSD assays, both performed admirably compared to other systems, particularly in detecting moderate-to-high concentrations of cytokines in spiked samples, but their performance characteristics were analyte-dependent (19). In a more recent publication, both LMX and MDS platforms were used to track 22 cytokine and cytokine receptor concentrations in subjects over time (3). While both assays provided good within-person correlation for up to 15 years, no effort was made to compare the platforms in terms of reliability in quantifying the same analytes.

Assay agreement in absolute quantification of the 16 shared cytokine analytes in human plasma was evaluated by agreement analysis based on Bland-Altman (20). The relative concentrations quantified by each assay are presented in a logarithmic format in which, for example, a skewing of the mean ratio by −1 or +1 indicates the mean cytokine quantity detected by the *y*-axis numerator (MSD) was either one-half or twice, respectively, of the analyte concentration categorized by comparator assay in the *y*-axis denominator (LMX). With the majority of shared analytes, the MSD assay categorized cytokine concentrations as less than 50% of those measured by the LMX platform. This may in part explain why the MSD assay missed detection of more cytokines in plasma samples than the LMX platform did, despite the MSD showing generally increased low-concentration sensitivity on calibration curves. Only in two instances (i.e., MIP-1α and MIP-1β) did LMX quantify mean analyte levels lower than did the MSD platform. In one instance (IL-17), the mean quantification ratio approximated 0, indicating similar categorization between samples. While the trends appear clear, this evaluation is tempered by the large standard deviations associated with the mean analyses for many analytes, which indicate large sample-to-sample variation in proportional quantification of those analytes by MSD and LMX. This would likely be clarified by evaluating a larger sample set and including more samples with increased analyte levels. One notable exception was with TNF-α, in which measured sample concentration ratios were much more uniformly distributed, resulting in a notably smaller standard deviation than the other analytes. Apparent proportional bias existed with one analyte, MIP-1β, where the mean quantification ratio increased as analyte concentration increased (20); however, interpretation of this trend is complicated by the large standard deviation observed for this analyte. There currently exist recommendations (28), but no clear standardized regulatory guidelines for validating multiplex assays for clinical use (29, 30). It is possible that standardization of different multiplex immunoplatforms by using identical reference proteins (e.g., World Health Organization or similar) and QC samples might reduce this variability (31).

Heatmaps were generated to visualize shared analyte concentrations measured by each assay in individual human plasma samples derived from healthy, diabetic, and high-PCT subjects. Whereas both assays showed clearly distinct cytokine expression differences between the high PCT group and the other two groups, results varied considerably among both analytes and distinct samples. Although general patterns emerged, such as higher relative cytokine expression in high-PCT (12, 16, 17) and possibly diabetic subjects (9) versus healthy control subjects within the LMX data, these observations were not consistent, possibly due to the low levels of cytokines measured in these samples.

The total time required to assay the 16 shared analytes was 5% less with the LMX versus the MSD assay, even though the instrument read time is much greater for the FLEXMAP 3D instrument than the MSD SECTOR Imager 6000 (≈20 min versus ≈2 min, respectively). This also takes into account that the MSD assay was performed using an alternative manufacturer-approved overnight capture antibody/sample incubation time instead of the usual 2-h incubation, so that sample incubation times were the same between platforms. Of total assay time, the labor time (i.e., hands-on and/or mandatory observation times) required to assess the 16 shared analytes by LMX was 36% less than that required by the MSD assays. However, it is important to point out that the MSD platform required handling 96-well plates from three different kits to accommodate sufficient analyte overlap with a single LMX plate because MSD plates are limited to a maximum of 10 analytes/well.

This study demonstrated the comparative performance and highlighted important differences of two multiplex platforms in a side-by-side comparison that simultaneously measured 16 shared cytokine analytes with known roles in pathogenesis. Study limitations included assessment of a limited number of human plasma samples, which was demonstrative but precluded some advanced statistical analyses. This study would have benefited from repetition using identical standard curve proteins and quality control samples, in addition to those provided with each kit. Although analyte diversity was good with both platforms, the potential cost, labor, and time savings associated with a larger multiplex set might be advantageous for certain applications, particularly when samples are rare or low-volume. Whereas both platforms demonstrated comparably excellent performance characteristics such as linearity, reproducibility and dynamic range, many more analytes in human plasma samples were below the level of detection by the MSD platform than the LMX system, even though the MSD calibration curves indicated significantly lower LLoQs for most of those analytes. This study demonstrates that assay and platform selection might depend upon the specific markers under investigation, time and cost considerations, and the anticipated concentration of target cytokines in prospective samples.

## Data Availability Statement

The raw data supporting the conclusions of this article will be made available by the authors, without undue reservation.

## Ethics Statement

The studies involving human participants were reviewed and approved by the University of Tübingen Institutional Review Board. All human samples were purchased from Central BioHub GmbH (Hennigsdorf, Germany). Analysis of these commercially available anonymous human plasma samples was exempted from IRB oversight requirements. Study performance complied with the tenets of the Declaration of Helsinki. Written informed consent for participation was not required for this study in accordance with the national legislation and the institutional requirements.

## Author Contributions

AG: methodology, formal analysis, investigation, and writing–original draft. MB: formal analysis, writing–original draft, and visualization. JG: conceptualization, formal analysis, and writing–reviewing and editing. TJ: conceptualization, formal analysis, writing – original draft, resources, supervision, and funding acquisition. NS-M: conceptualization, methodology, resources, writing-original draft, writing – reviewing and editing, visualization, supervision, project administration, and funding acquisition. All authors read and approved the final manuscript version.

## Conflict of Interest

This study was funded by Luminex Corporation, Austin, TX, United States. Some assay kits were provided by Merck EMD Millipore, Billerica, MA, United States. The Natural and Medical Sciences Institute at the University of Tübingen is involved in applied research projects as a fee for services with Luminex. TJ is a scientific advisor for Luminex. NS-M has previously been an invited speaker at Luminex user meetings. The remaining authors declare that the research was conducted in the absence of any commercial or financial relationships that could be construed as a potential conflict of interest.

## Footnotes

1 https://www.r-project.org

## Supplementary Material

Supplementary Figure 1 Time and motion analysis. The total time required for performing the LMX 21-plex and three MSD 10-plex assays was approximately 20.5 h and 21.5 h for the LMX and MSD assays, respectively (includes overnight primary incubation). Of this time, hands-on labor time accounted for approximately 1.6 h for LMX and 2.5 h for the MSD assays. For MSD assays, we opted to use the manufacturer-approved overnight sample incubation instead of the standard 2-h incubation, to better match total assay times. The MSD kit user manuals also indicate that this extended sample incubation may increase assay sensitivity. We acknowledge that the difference in labor time was likely due to the necessity of using multiple 10-plex MSD kits (their maximum number of analytes then available per kit) compared to only a single 21-plex Luminex kit to achieve desired analyte overlap. The MSD assay has the benefit of a 70-s plate reading time whereas the LMX assay requires 20 min per plate.

## Abbreviations

CV: coefficient of variation
LLoQ: lower limit of quantification
LMX: Luminex bead-based fluorescence multiplex assay
LoD: limit-of-detection
MSD: Meso Scale Discovery planar electrochemiluminescence multiplex assay
QC: quality control sample
ULoQ: upper limit of quantification.
