# Supplementary figures and images for: Comparison of Bead-Based Fluorescence Versus Planar Electrochemiluminescence Multiplex Immunoassays for Measuring Cytokines in Human Plasma
Source: Front Immunol. 2020 Sep 24;11:572634. doi: 10.3389/fimmu.2020.572634 (PMC7546899; doi:10.3389/fimmu.2020.572634)

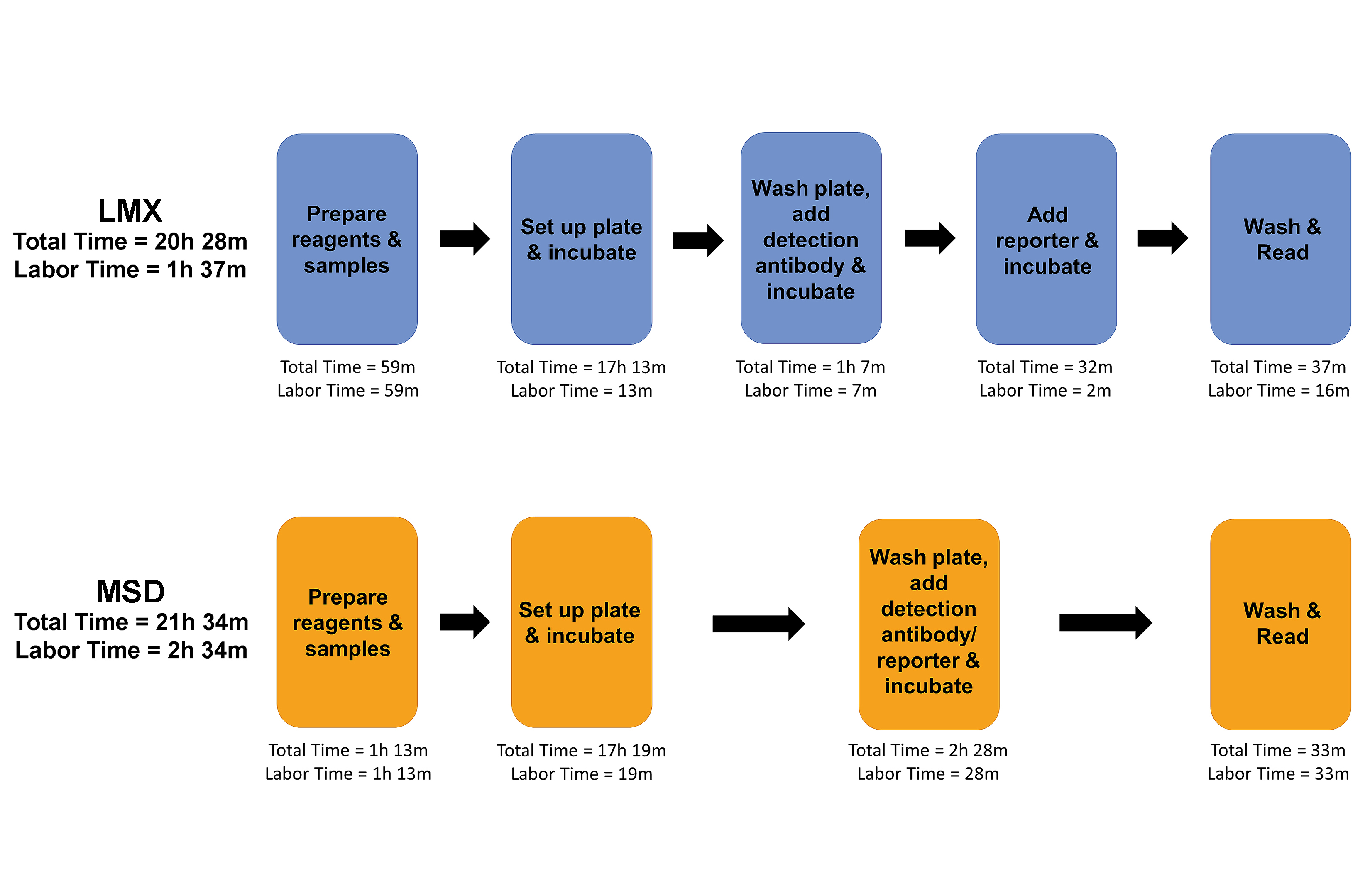

Supplement: Supplementary Figure 1 — Time and motion analysis. The total time required for performing the LMX 21-plex and three MSD 10-plex assays was approximately 20.5 h and 21.5 h for the LMX and MSD assays, respectively (includes overnight primary incubation). Of this time, hands-on labor time accounted for approximately 1.6 h for LMX and 2.5 h for the MSD assays. For MSD assays, we opted to use the manufacturer-approved overnight sample incubation instead of the standard 2-h incubation, to better match total assay times. The MSD kit user manuals also indicate that this extended sample incubation may increase assay sensitivity. We acknowledge that the difference in labor time was likely due to the necessity of using multiple 10-plex MSD kits (their maximum number of analytes then available per kit) compared to only a single 21-plex Luminex kit to achieve desired analyte overlap. The MSD assay has the benefit of a 70-s plate reading time whereas the LMX assay requires 20 min per plate. [file Image_1.jpg]
