# Supplementary material for: Comparison of Bead-Based Fluorescence Versus Planar Electrochemiluminescence Multiplex Immunoassays for Measuring Cytokines in Human Plasma
Source: Front Immunol. 2020 Sep 24;11:572634. doi: 10.3389/fimmu.2020.572634 (PMC7546899; doi:10.3389/fimmu.2020.572634)
Supplement: Supplementary file 2 [file Table_1.DOCX]

**Supplementary Table 1.** Limit of Detection, Lower Limit of Quantification, and Dynamic Range of analytes measured by the bead-based fluorescence (LMX) and planar electrochemiluminescence (MSD)

multiplex assays.

|  | **LMX** | | | **MSD** | | |
| --- | --- | --- | --- | --- | --- | --- |
| **16 Shared**  **Analytes** | **LoD (pg/mL)** | **LLoQ  (pg/mL)** | **Dynamic Range (pg/mL)** | **LoD  (pg/mL)** | **LLoQ (pg/mL)** | **Dynamic Range (pg/mL)** |
| GM-CSF | 5.91 | 5.91 | 5.91‒20,000 | 0.38 | 1.05 | 1.05‒4,280 |
| IFN-γ | 2.01 | 2.44 | 2.44‒10,000 | 1.43 | 1.43 | 1.43‒5,080 |
| IL-1β | 0.61 | 1.95 | 1.95‒8,000 | 0.10 | 0.56 | 0.56‒2,296 |
| IL-2 | 2.41 | 2.41 | 2.41‒8,000 | 0.22 | 1.37 | 1.37‒5,600 |
| IL-4 | 8.21 | 8.21 | 8.21‒30,000 | 0.03 | 0.21 | 0.21‒872 |
| IL-5 | 0.88 | 1.95 | 1.95‒8,000 | 0.42 | 0.81 | 0.81‒3,320 |
| IL-6 | 0.59 | 0.73 | 0.73‒3,000 | 0.09 | 0.73 | 0.73‒2,992 |
| IL-7 | 1.16 | 1.46 | 1.46‒6,000 | 0.28 | 0.83 | 0.83‒3,384 |
| IL-8 | 0.78 | 1.22 | 1.22‒5,000 | 0.08 | 0.54 | 0.54‒2,212 |
| IL-10 | 3.77 | 5.86 | 5.86‒24,000 | 0.08 | 0.37 | 0.37‒1,504 |
| IL-12 p70 | 0.71 | 1.95 | 1.95‒8,000 | 0.10 | 0.54 | 0.54‒2,204 |
| IL-13 | 2.19 | 2.19 | 2.19‒4,000 | 0.41 | 0.49 | 0.49‒2,012 |
| IL-17 | 1.63 | 2.93 | 2.93‒12,000 | 0.87 | 6.09 | 6.09‒24,960 |
| MIP-1α | 18.90 | 18.90 | 18.90‒5,000 | 7.25 | 7.25 | 7.25‒4,560 |
| MIP-1β | 7.09 | 7.09 | 7.09‒15,000 | 0.90 | 1.15 | 1.15‒4,720 |
| TNF-α | 0.63 | 1.71 | 1.71‒7,000 | 0.16 | 0.32 | 0.32‒1,292 |
| **Unshared**  **Analytes** | **LoD (pg/mL)** | **LLoQ  (pg/mL)** | **Dynamic Range (pg/mL)** | **LoD  (pg/mL)** | **LLoQ (pg/mL)** | **Dynamic Range (pg/mL)** |
| Fractalkine | 52.20 | 73.20 | 73.20‒300,000 | ‒ | ‒ | ‒ |
| IL-21 | 1.26 | 1.26 | 1.26‒4,000 | ‒ | ‒ | ‒ |
| IL-23 | 56.30 | 56.30 | 56.30‒130,000 | ‒ | ‒ | ‒ |
| ITAC | 2.40 | 5.86 | 5.86-24,000 | ‒ | ‒ | ‒ |
| MIP-3a | 3.05 | 3.05 | 3.05‒10,000 | ‒ | ‒ | ‒ |
| Eotaxin-1 | ‒ | ‒ | ‒ | 4.61 | 4.61 | 4.61‒6,880 |
| Eotaxin-3 | ‒ | ‒ | ‒ | 10.30 | 10.30 | 10.30‒24,960 |
| IL-1a | ‒ | ‒ | ‒ | 3.50 | 3.50 | 3.50‒1,816 |
| IL-8 HA | ‒ | ‒ | ‒ | 55.30 | 77.80 | 77.80‒318,800 |
| IL-12 p40 | ‒ | ‒ | ‒ | 0.61 | 3.56 | 3.56‒14,600 |
| IL-15 | ‒ | ‒ | ‒ | 0.22 | 0.81 | 0.81‒3,316 |
| IL-16 | ‒ | ‒ | ‒ | 0.54 | 2.58 | 2.58‒10,560 |
| IP-10 | ‒ | ‒ | ‒ | 0.25 | 2.30 | 2.30‒9,440 |
| MCP-1 | ‒ | ‒ | ‒ | 0.21 | 0.55 | 0.55‒2,248 |
| MCP-4 | ‒ | ‒ | ‒ | 2.15 | 2.15 | 2.15‒2,896 |
| MDC | ‒ | ‒ | ‒ | 13.10 | 13.10 | 13.10-47,200 |
| TARC | ‒ | ‒ | ‒ | 1.38 | 1.74 | 1.74‒7,120 |
| TNF-beta | ‒ | ‒ | ‒ | 0.14 | 0.69 | 0.69‒2,844 |
| VEGF | ‒ | ‒ | ‒ | 0.68 | 1.09 | 1.09‒4,480 |

LoD = Limit of Detection, LLoQ = Lower Limit of Quantitation, “‒” indicates this analyte was not included in that platform.
