# Supplementary material for: Comparison of Bead-Based Fluorescence Versus Planar Electrochemiluminescence Multiplex Immunoassays for Measuring Cytokines in Human Plasma
Source: Front Immunol. 2020 Sep 24;11:572634. doi: 10.3389/fimmu.2020.572634 (PMC7546899; doi:10.3389/fimmu.2020.572634)
Supplement: Supplementary file 3 [file Table_2.DOCX]

**Supplementary Table 2.** Comparison of dynamic ranges of analytes shared by Luminex bead-based fluorescence (LMX) and Meso Scale Discovery electrochemiluminescence (MSD) platforms, presented as “fold of LLoQ”. Standard 1 (STD1) refers to the highest concentration in the standard curve for that assay’s individual analytes. The two platforms showed dynamic ranges that were within 15% of one another for 11 of 16 shared analytes. In the remaining 5 shared analytes, the LMX/MDS dynamic range ratios varied from 42% (MIP-1α) to 83% (GM-CSF).

| **Shared**  **Analytes** | **LMX STD1 (pg/mL)** | **LMX Dynamic Range**  **(fold of LLoQ)** | **MSD STD1 (pg/mL)** | **MSD Dynamic Range**  **(fold of LLoQ)** | **Dynamic Range Ratio**  **LMX/MSD** |
| --- | --- | --- | --- | --- | --- |
| **GM-CSF** | 20,000 | 3,382 | 4,280 | 4,096 | **0.83** |
| **IFN-γ** | 10,000 | 4,096 | 5,080 | 3,558 | **1.15** |
| **IL-1β** | 8,000 | 4,096 | 2,296 | 4,096 | **1.00** |
| **IL-2** | 8,000 | 3,318 | 5,600 | 4,096 | **0.81** |
| **IL-4** | 30,000 | 3,656 | 872 | 4,096 | **0.89** |
| **IL-5** | 8,000 | 4,096 | 3,320 | 4,096 | **1.00** |
| **IL-6** | 3,000 | 4,096 | 2,992 | 4,096 | **1.00** |
| **IL-7** | 6,000 | 4,096 | 3,384 | 4,096 | **1.00** |
| **IL-8** | 5,000 | 4,096 | 2,212 | 4,096 | **1.00** |
| **IL-10** | 24,000 | 4,096 | 1,504 | 4,096 | **1.00** |
| **IL-12 p70** | 8,000 | 4,096 | 2,204 | 4,096 | **1.00** |
| **IL-13** | 4,000 | 1,827 | 2,012 | 4,096 | **0.45** |
| **IL-17** | 12,000 | 4,096 | 24,960 | 4,096 | **1.00** |
| **MIP-1α** | 5,000 | 264 | 4,560 | 629 | **0.42** |
| **MIP-1β** | 15,000 | 2,117 | 4,720 | 4,096 | **0.52** |
| **TNF-α** | 7,000 | 4,096 | 1,292 | 4,096 | **1.00** |

LLoQ = Lower limit of quantification
